# Classification of ADHD children through multimodal magnetic resonance imaging

**DOI:** 10.3389/fnsys.2012.00063

**Published:** 2012-09-03

**Authors:** Dai Dai, Jieqiong Wang, Jing Hua, Huiguang He

**Affiliations:** ^1^Neural Imaging Computation and Analysis Group, State Key Laboratory of Management and Control for Complex Systems, Institute of Automation, Chinese Academy of SciencesBeijing, China; ^2^Graphics and Imaging Laboratory, Department of Computer Science, Wayne State UniversityDetroit, MI, USA

**Keywords:** attention deficit/hyperactivity disorder, ADHD-200 competition, resting-state functional connectivity, support vector machine, multi-kernel learning

## Abstract

Attention deficit/hyperactivity disorder (ADHD) is one of the most common diseases in school-age children. To date, the diagnosis of ADHD is mainly subjective and studies of objective diagnostic method are of great importance. Although many efforts have been made recently to investigate the use of structural and functional brain images for the diagnosis purpose, few of them are related to ADHD. In this paper, we introduce an automatic classification framework based on brain imaging features of ADHD patients and present in detail the feature extraction, feature selection, and classifier training methods. The effects of using different features are compared against each other. In addition, we integrate multimodal image features using multi-kernel learning (MKL). The performance of our framework has been validated in the ADHD-200 Global Competition, which is a world-wide classification contest on the ADHD-200 datasets. In this competition, our classification framework using features of resting-state functional connectivity (FC) was ranked the 6th out of 21 participants under the competition scoring policy and performed the best in terms of sensitivity and J-statistic.

## Introduction

Attention deficit/hyperactivity disorder (ADHD), one of the most commonly diagnosed childhood behavioral disorders, is characterized by inappropriate inattention, impulsivity, and hyperactivity. ADHD affects at least 5% of school-age children, making them difficult to control their behaviors or focus their attentions. These symptoms may persist into adulthood and result in a lifelong impairment (Biederman et al., 2000). In spite of a large amount of research efforts, the community has not yet comprehensively understood the pathology of ADHD. Moreover, the current practice in the diagnosis of ADHD is mainly according to the levels of symptoms listed in DSM-IV (American Psychiatric Association, 1994), and the diagnosis is usually conducted by the parents or teachers, which is unfortunately subjective. In fact, it is very difficult to draw a line between the normal levels of the ADHD symptoms and the clinically significant levels that require interventions. Thus, further studies on objective diagnosis of ADHD are of great significance.

Recently, structural MRI (sMRI) and functional MRI (fMRI) have been widely used to examine the brain of ADHD patients, and various abnormalities have been reported. Studies using sMRI showed a totally decreased cerebral volume of 3–5% (Seidman et al., 2005; Valera et al., 2007), and abnormalities in several specific brain regions such as lateral prefrontal cortex, cingulate cortex, striatum, cerebellum, and callosum (Semrud-Clikeman et al., 2000; Overmeyer et al., 2001; Kates et al., 2002; Seidman et al., 2005; Valera et al., 2007). As for fMRI studies, abnormal brain activations were found in task-related experiments on the dorsal anterior cingulate cortex (dACC), the ventrolateral prefrontal cortex (VLPFC), and the putamen (Bush et al., 1999; Teicher et al., 2000; Durston et al., 2003). Resting-state fMRI (rs-fMRI) was also used in ADHD studies and abnormalities were found in prefrontal cortex, inferior frontal cortex, sensorimotor cortex, anterior cingulated cortex, putamen, temporal cortex, and cerebellum (Cao et al., 2006; Tian et al., 2006; Zang et al., 2007; Liu et al., 2010). In addition, Castellanos et al. (2008) found ADHD-related decreases of functional connectivity (FC) between anterior cingulate and precuneus/posterior cingulate cortex regions, as well as between precuneus and other default-mode network components, including ventromedial prefrontal cortex and portions of posterior cingulate cortex.

Although studies using between-group statistics may indicate the abnormal regions of ADHD patients, it has been argued that between-group analysis might be less useful for automatic diagnosis (Seidman et al., 2004). The wide utilization of modern machine learning techniques in neuroimaging community makes it possible for researchers to discover clinical biomarkers of the diseases and develop automatic diagnosis systems. Many efforts have been made to use sMRI or fMRI to predict patients of Alzheimer's disease and schizophrenia (Fan et al., 2007; Hinrichs et al., 2009, 2011). However, few studies have been conducted to make prediction of ADHD. Zhu et al. (2008) trained a classifier of principal-components-analysis-based (PCA-based) fisher-discriminant-analysis (FDA) using features of regional homogeneity (ReHo) in their study. They obtained as high as 85% leave-one-out cross validation (LOOCV) accuracy, but the samples for experiments were quite small (only 24 subjects).

In order to accelerate the understanding of ADHD and to obtain objective diagnosis methods, the ADHD-200 Consortium publically released the ADHD-200 Samples and held the ADHD-200 Global Competition in 2011. The aim of the contest was to have participants develop and train an image-based diagnosis classifier to predict ADHD-Combined type (ADHD-C), ADHD-Inattention type (ADHD-I) and typically developing control (TDC), based on the 776 samples in the ADHD-200 datasets. Then a test set of 195 unlabeled samples will be used to evaluate the performance of the classifiers developed by the participants. Please refer to the ADHD-200 website (fcon_1000.projects.nitrc.org/indi/adhd200/) for the scoring policy and more details.

In our work, we conducted extensive experiments on the features which were extracted from sMRI and rs-fMRI, as well as the combination of both, namely, multi-modal features. We finally selected the FC as the feature to train a support vector machine (SVM) in the contest, because the best cross validation (CV) accuracy was achieved when using this feature. In the final results released by ADHD-200 consortium, we were ranked the 6th out of 21 participants under the competition scoring policy, and our method performed the best in terms of sensitivity and J-statistic. In this paper, we list and compare the performances of all kinds of features to provide a comprehensive understanding of potential useful information related to ADHD diagnosis. The main contribution of this work is that we proposed an automatic classification framework for ADHD and achieved good results in ADHD-200 Global Competition, which constituted a useful exploration for ADHD classification.

The remainder of this paper is organized as follows. The subject selection, feature extraction, feature selection, and classification methods are introduced in “Materials and Methods” section. “Results” section shows the performance and results of the proposed methods. The discussion and conclusion of this paper are given in “Discussion” section.

## Materials and methods

### Datasets and subjects

The datasets for the competition consist of structural and rs-fMRI of 776 labeled subjects for training and 195 unlabeled subjects for testing. The labeled training set contains 285 individuals diagnosed with ADHD and 491 typical developed children (TDC). These samples are collected from eight sites, using different scanners and scanning parameters. Not all the subjects in ADHD-200 datasets are available or suitable for the studies, e.g., images of some subjects are of low quality, making them prone to fail in the preprocessing procedure and produce unconvincing features for classification. Thus, we exclude several subjects before our experiments according to the following schemes: (1) All the images are examined after the scanning and marked with a label of quality control in the phenotypic key. We only retain subjects whose images have a quality control of 1; (2) The subjects of ADHD-Hyperactive type (ADHD-HI) do not contribute to the classifier due to the small number of samples and no requirement to distinguish them from others in the contest. Thus, we exclude all subjects of ADHD-HI type; (3) We exclude subjects whose images fail in preprocessing or feature extraction procedure. In total, 152 subjects out of 776 are excluded and 624 remain. The number of subjects from each site is listed in Table 1, and the demographic information is shown in Table 2.

**Table 1:** **Subjects selected from different sites**.

	**Total**	**TDC**	**ADHD-C**	**ADHD-I**
PEKING	181	108	27	46
KKI	77	57	15	5
NI	32	22	10	0
NYU	179	87	60	32
OHSU	60	33	15	12
Pittsburgh	59	59	0	0
WashU	36	36	0	0
Total	624	402	127	95

**Table 2:** **The demographic information of selected subjects**.

	**Total**	**TDC**	**ADHD-C**	**ADHD-I**
Sex(M/F)	381/242	208/194	104/22	69/26
Age	12.16 ± 3.20	12.47s ± 3.36	11.32 ± 2.98	12.02 ± 2.52

### Image preprocessing and original feature extraction

In order to obtain potential features related to ADHD, we employ different image processing techniques to extract multimodal features. For sMRI, cortical thickness (CT), and gray matter probability (GMP) are extracted while for rs-fMRI, ReHo, and FC are extracted. The preprocessing steps and extraction of features are detailed in the following.

#### Cortical thickness

Cortical surface is reconstructed by FreeSurfer (v5.0.0, surfer.nmr.mgh.harvard.edu/) using the methods proposed by Dale et al. (1999) and Fischl et al. (1999), and CT on each vertex is measured. First, all T1-weighted images are corrected for intensity non-uniformity and registered into a template. Then the gray matter (GM) and white matter (WM) and subcortical tissues are segmented. A smoothly tessellated cortical surface is constructed for each hemisphere and automatic correction is preformed to remove topological defects. After that, the pial surface and GM/WM surface are built and the shortest distances from pial surface to GM/WM surface and from GM/WM surface to pial surface are calculated, respectively, on each vertex. Finally, CT on each vertex is obtained as the mean value of these two distances and a surface-based diffusion smoothing with 20 mm full-width-at-half-maximum (FWHM) is conducted to enhance the statistical performance.

#### Gray matter probability

All the structural images are preprocessed using voxel-based morphometry (VBM) toolbox in Statistical Parametric Mapping (SPM8, www.fil.ion.ucl.ac.uk/spm). First the original anatomical images are segmented into GM, WM, and cerebrospinal fluid images (CSF). Then the segmented images are registered to the Montreal Neurological Institute (MNI) template using 12-parameter affine transformation and non-linear deformation with a warp frequency cutoff at 25. A modulation process is also employed, which scales the final GM images by the amount of contraction required to warp the images to the template. The final result is GM volume maps for each subject, where the total amount of GM remains the same as in the original images. Finally, the normalized maps are smoothed using an 8 mm isotropic Gaussian kernel to improve signal-to-noise ratio and facilitate comparison across subjects. We use only GMP as the features.

#### Regional homogeneity

Some image preprocessing steps should be conducted before features of rs-fMRI are extracted. The first 10 volumes of each functional time series are discarded for participant adaptation to the scanning. Then the image data are temporally realigned to remove time delay between different slices, and spatially realigned to remove head motions. If the head motions of a session are over the threshold of 2 mm (Power et al., 2012), this session of the subject will be abandoned and other sessions are used. We further spatially normalize the realigned images to the MNI template. Considering the sMRI images of subjects are offered, T1 image unified segmentation (Ashburner and Frison, 2005) is applied to normalize the realigned images. Then the normalized images are resampled to voxels of 3 × 3 × 3 mm^3^. At last, linear drift detrend and temporal band-pass filtering (0.01 < f < 0.08 Hz) (Fox et al., 2005; Liang et al., 2006) are performed to reduce low-frequency drift and high-frequency noise using the rs-fMRI data analysis toolkit (Song et al., 2011) (REST V1.6, www.restfmri.net/).

ReHo was originally proposed by Zang et al. (2004). It uses Kendall's coefficient of concordance (KCC) to measure the regional synchrony for the given voxel and its *K*-1 nearest neighbors, and is calculated as

(1) $$W=\frac{\sum_{i}R_{i}^{2}-n\cdot {\bar{R}}^{2}}{\frac{1}{12}K^{2}\left(n^{3}-n\right)}$$

where *W* is the KCC of the given voxel, ranging from 0 to 1; *K* = 27 is the number of neighborhood voxels and *n* is the number of time points; *R*_*i*_ is the sum of *K* voxels on the *i*-th time point; $\bar{R}$ is the mean of the R_*i*_'s. ReHo is calculated on each voxel of the whole brain to form a ReHo map using REST toolkit. A smoothing process (8 mm FWHM of Gaussian kernel) is conducted on the ReHo map to reduce noise.

#### Functional connectivity

FC is measured as the correlation coefficient of time courses of any two voxels or ROIs. In this study, we mainly use ROI-based FC due to the low computational complexity. We utilize the CC400 atlas Craddock et al. (2012), which is used in Athena pipeline of ADHD preprocessed data (neurobureau.projects.nitrc.org/ADHD200/Introduction.html), to extract time courses of 351 ROIs. Then ROI-based FC is calculated for each subject as follow

(2) $$FC\left(i,j\right)=\frac{\sum \left(X_{i}-\bar{X_{i}}\right)\left(X_{j}-\bar{X_{j}}\right)}{\sqrt{\sum \left(X_{i}-\bar{X_{i}}\right)\sum \left(X_{j}-\bar{X_{j}}\right)}}$$

where *X*_*i*_ is the time courses of the *i*-th ROI; $\bar{X_{i}}$ is the average time courses of the *i*-th ROI; and *FC*(*i, j*) presents the connection weight between the *i*-th and the *j*-th ROI. The calculated FC is pruned using an absolute threshold of 0.05 in order to reduce noise.

### Feature selection

The dimensionality of original brain features is usually much higher than the number of samples, which cannot be directly used to train classifier considering overfitting problem and computational complexity. Thus, dimensionality reduction is required to improve the performance of the classifier. Feature selection is a kind of commonly used dimensionality reduction methods, as opposed to feature extraction such as PCA, in which new low-dimensional embedding is produced using the original features. In this study, we use a hybrid feature selection method which combines filter-based and wrapper-based methods (Kohavi and John, 1997).

The discriminative power of a feature can be estimated using within-class sum of squares (WSS) and between-class sum of squares (BSS). A smaller WSS and a larger BSS usually mean that a feature is more prone to be distinguished. Thus, the ratio of BSS to WSS is used to rank features. Specifically, donating *f*_*ij*_ as the value of the *i*-th feature of the *j*-th sample and *c* is the group label (+1 or −1), the ranking score of the *i*-th feature is defined as

(3) $$r_{i}=\frac{BSS_{i}}{WSS_{i}}=\frac{\sum_{j}\sum_{c}I\left(y_{j}=c\right){\left(\bar{f_{ic}}-\bar{f_{i}}\right)}^{2}}{\sum_{j}\sum_{c}I\left(y_{j}=c\right){\left(f_{ij}-\bar{f_{ic}}\right)}^{2}}$$

where $\bar{f_{ic}}$ is the mean value of the *i*-th feature across subjects in class *c* and $\bar{f_{i}}$ is the mean value of the *i*-th feature across all subjects; the index function *I*(*y*_*j*_ = *c*) equals 1 if the *j*-th subject belongs to group *c* and equals 0 otherwise.

However, the above filter method computes the ranking scores independently for each feature, which does not take into account the relationship (redundant or complementary) between features. In other words, it does not optimize the feature subset as a whole for a specific classification problem. Thus, the selected features might not be the optimal feature subset for the classification of ADHD. In fact, features with weak discriminative power may contribute to the performance of classification if they are complementary to others, while those with strong power may affects the performance if they are redundant.

To avoid this problem, we employ a wrapper-based method for feature subset selection utilizing SVM based on recursive feature elimination which is named SVM-RFE (Guyon et al., 2002). In this algorithm, SVM is trained iteratively using selected feature subset. In each iteration, the ranking score for each feature in the selected feature subset is calculated during the SVM training process (e.g., for linear SVM we can simply consider the ranking score of features as the *w* that satisfies *y* = *w* · *x* + *b*, however, the SVM-RFE algorithm uses the radial basis function kernel, which is slightly complicated for estimation of the score). A portion of features with small score are eliminated in each iteration of SVM training until the classification accuracy is over a set threshold, or the number of remaining features in the selected subset is smaller than a set value. Note that SVM-RFE uses the accuracy of CV to estimate the goodness of feature subset, which may avoid the overfitting problem. Thus, we first use the ratio of BSS/WSS to filter most features with little discriminative power, and then use SVM-RFE for further refined feature selection. This will ensure the selection optimal feature subset at relatively low computational cost.

### Classification methods

**Figure 1:**
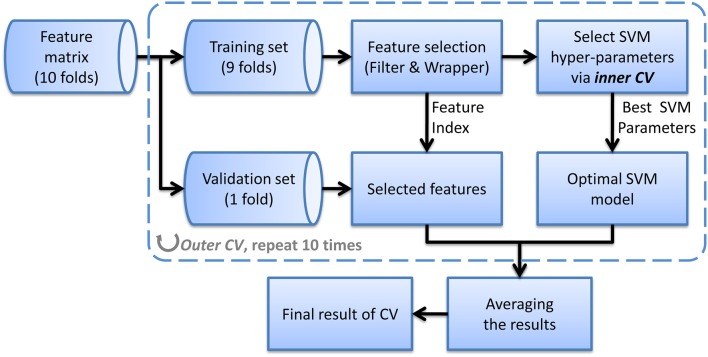
**The flow chart of the nested CV classification method using a single feature**.

In our framework, SVMs with radial basis function (RBF) kernel are used for classification. Let *x*_1_, *x*_2_ be the feature vectors, and RBF kernel is defined as

(4) $$K\left(x_{1},x_{2}\right)=\exp\left(-\frac{{\left\|x_{1}-x_{2}\right\|}^{2}}{2\sigma^{2}}\right)$$

where σ is the width of the kernel. The hyper-parameters of SVM such as the penalized coefficient and the kernel width should be carefully tuned to obtain the optimal SVM model. We apply an automatic searching method which uses grid search to tune parameters and CV to evaluate the goodness of them. Usually, this CV for parameter tuning is named inner CV because it is nested in another CV called outer CV, which is used for evaluating the generalization of the method (see Wilson et al., 2009 for more details of nested CV). We use a 10-fold outer CV in this study, that is, in each fold of outer CV, one tenth samples from each class are kept out for validation and the remainder are used for selecting features and training classifier (including selecting hyper-parameters). Then the held-out samples are validated using the selected features and parameters which are obtained in the training process. After all samples have been validated once, we calculate the average CV accuracy and consider it as the estimation of generalization. This nested CV method can yield an unbiased assessment of the classification method and prevent overestimation. Figure 1 shows the flow chart of the evaluation method we used for nested CV.

In addition to using SVM as classifier, we also attempt to apply multi-kernel learning (MKL) to integrate multi-modal features (Sonnenburg et al., 2006; Hinrichs et al., 2011). In MKL framework, each kind of feature is assigned an independent kernel and different parameters, and the MLK algorithm can automatically search the optimal combination of the kernel matrix of these features to form an integrated kernel matrix (assigning weight to each kernel matrix), which could be better than any of single kernels. The output of MKL is defined as

(5) $$y_{i}=\sum_{k}\beta_{k}\left(\sum_{j}\lambda_{j}^{k}y_{j}K_{k}\left(x_{j}^{k},x_{i}^{k}\right)\right)+b$$

where *k* denotes the *k*-th kind of feature; *K*_*k*_ is the kernel matrix; β_*k*_ is the sub-kernel weight; λ^*k*^_*j*_ is the Lagrange parameters and *x*^*k*^_*i*_ is the features of the *i*-th subject. Although MKL is theoretically better, it has more parameters to be tuned. Thus, the training process will be much more time-consuming, and sub-optimal model tends be obtained which might affect the effectiveness of MKL.

### Implementation

Our classification framework and validation experiments was implemented in Matlab using an interface to the LIBSVM (v3.1.2, www.csie.ntu.edu.tw/~cjlin/libsvm/) for the SVM classifier and Shogun (v1.1.0, www.shogun-toolbox.org/) for the MKL framework. The main source code for this work will be made available on the website of NICA group (nica.ia.ac.cn/research/t20120712_1145.htm?lang=en). Nonetheless, please note that you have to prepare the original image features by yourself.

## Results

### Classification results using a single feature

A hierarchical 2-class classifier is used, which first classify subjects into ADHD group and TDC group, and then into ADHD subtype. The 10-fold CV classification results using a single feature are listed in Table 3, in which we compare the CV accuracy using CT, GMP, ReHo, or ROI-based FC as the feature to train the classifier. In addition, sensitivity (true positive rate, TPR) and specificity (true negative rate, TNR) are calculated to evaluate the classification performance without considering class distribution. J-statistic is a combined measure of sensitivity and specificity and is calculated as *sensitivity + specificity* − 1. J-statistic is used by the ADHD-200 Consortium to compare the competition results of participating groups (fcon_1000.projects.nitrc.org/indi/adhd200/results.html), although it is not generally to be recommended (Youden, 1950). Similar to J-statistic, F-score is another measure considering both recall and precision, which is commonly used in information retrieval. F_1_-score can also be calculated in terms of type I and type II errors as

**Table 3:** **The 10-fold CV classification results using a single kind of feature and multimodal features**.

**Feature**	**CV Accuracy (2-class)**	**Sensitivity/Specificity**	**J-statistic**	**F_1_-score**	**AUC**	**CV Accuracy (3-class)**
CT	61.38%	18.47/85.07%	0.0353	0.2539	0.5870	49.12%
GMP	64.90%	**45.50**/75.62%	**0.2112**	**0.4798**	**0.6787**	**56.87%**
ReHo	**65.87%**	22.52/**89.80%**	0.1232	0.3195	0.5982	56.15%
FC	62.02%	41.89/73.13%	0.1502	0.4397	0.6365	54.92%
MKL	67.79%	38.29 / 84.08%	0.2237	0.4582	0.7068	57.71%

The bold font means the best performance using a single feature. The performance of MKL is listed in the bottom and is better than that using a single feature.

(6) $$F_{1}=\frac{2\cdot TPos}{2\cdot TPos+FNeg+FPos}$$

where *TPos*, *FNeg*, and *FPos* are the number of true positive, false negative, and false positive. The larger J-statistic and F-score, the better performance of a classifier. Besides, area under ROC curve (AUC) is an evaluation measure derived from receiver operating characteristic (ROC) curve. The ROC curve is a graph evaluation method which can illustrate the performance of a binary classifier as its decision threshold is varied. When the decision threshold of a classifier varies, sensitivity and specificity also change. ROC curve is created by plotting *sensitivity* and 1–*specificity* at different thresholds. A larger AUC commonly indicates a better classifier.

**Figure 2:**
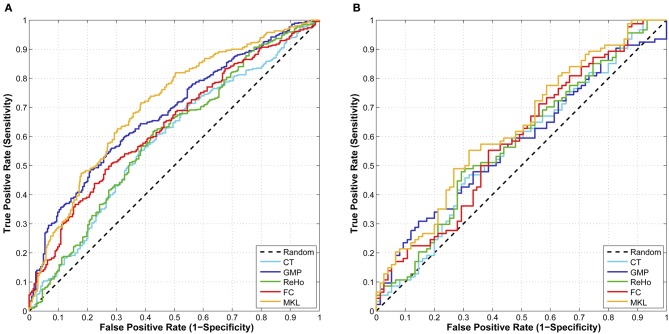
**(A)** The ROC curve of CV classification on training set of 624 subjects; **(B)** The ROC curve of classification on test set of 169 subjects.

From the results listed in Table 3, we find that classification using features of ReHo achieves the best CV accuracy. However, it does not work well in terms of J-statistic, F_1_-score and the area under ROC curve (AUC). This is directly caused by imbalanced sensitivity and specificity and might be implicitly caused by the imbalance of class distribution which we will discuss later. Classifiers using features of GMP and ROI-based FC both achieve good CV accuracy and AUC, as well as more balanced sensitivity and specificity (a higher J-statistic or F_1_-score). Figure 2A shows the ROC curve of the CV classification using different kinds of features.

We also test the classifier built using a single feature on the test set of 169 samples (the labels for 26 samples from Brown University are not available) and the classification results are summarized in Table 4. Classifier using feature of ROI-based FC achieves the best results in terms of almost all the criteria. Although the classification results on test set are a little worse than the CV results on training set, the difference between them is not significant. This suggests that our CV classification framework could estimate the performances with little bias.

**Table 4:** **The classification results on test set using a single kind of feature**.

**Feature**	**Accuracy (2-class)**	**Sensitivity/Specificity**	**J-statistic**	**F_1_-score**	**AUC**	**Accuracy (3-class)**
CT	55.62%	22.67/81.91%	0.0458	0.3119	0.5212	44.1%
GMP	56.80%	34.67/74.47%	0.0917	0.4160	0.6065	52.6%
ReHo	56.80%	17.57/90.30%	0.0788	0.2913	0.5480	48.1%
FC	**59.17%**	44.00/71.28%	**0.1528**	**0.4889**	**0.6187**	**53.2%**
MKL	61.54%	41.33 / 77.66%	0.1899	0.4882	0.6288	54.1%

The bold font means the best performance using a single feature. The performance of MKL is listed in the bottom and is better than that using a single feature.

### Classification results using MKL

In order to integrate the above four kinds of features, an MKL classifier is trained. To reduce computational complexity, we skip the feature selection and parameter tuning steps and directly use the same feature subset and parameters in classifications using a single feature, which might not be the optimal ones for MKL classifier. However, the classification results on both training set and test set exceed the best ones of classification using only a single feature. This suggests that MKL that integrates multimodal features is potentially powerful, although it is more difficult to choose hyper-parameters and is more time consuming. The classification results using MKL are also listed in Tables 3 and 4, and the ROC curve can be found in Figure 2. We can find that the ROC curve of MKL classifier is better (nearer to northwest) than other classifier using a single feature, and the AUC of the former is larger than the latter.

## Discussion

### Classification using multi-site datasets

Because of the difficulty of collecting samples, datasets in imaging study are usually quite small in the neuroimaging community. One hundred samples are already quite large dataset and cost thousands of dollars and several months to collect. Thus, opening or exchanging neuroimaging data can produce much larger datasets, making the researches more reliable and convincing. This will benefit the whole community as the ADHD-200 datasets did. However, datasets from different sites usually vary in their scanning device or parameters, and the race of subjects may also be different. These factors might result in different baselines of extracted features. Although some studies argue that multi-site datasets do not significantly affect their results (Pardoe et al., 2008; Bendfeldt et al., 2012), we find that these studies have some obvious limitations: (1) The number of scanning sites is limited—only two or three, while ADHD-200 Sample have eight scanning sites; (2) All datasets have both patients and normal control, while in ADHD-200 Sample 2 sites have only normal controls. (3) They implement VBM analysis on multi-site datasets rather than classification. Thus, their conclusions may not be applied to our study.

In our experiment, we find that the impacts of multi-site datasets on each kind of feature are not similar. For instance, we also use scale-invariant feature transform (SIFT) to extract features for classification and we find that these features can even distinguish subjects from different sites, which means that they can hardly be used directly to predict ADHD patients because of the evidently distinct baseline. We consider that several potential methods may alleviate this problem. (1) The first is to construct a regression model with respect to these factors. Nonetheless, this method might produce more uncertainty, because the impacts of factors such as scanning parameters or races on the human brains are much more complicated than age or gender. A simple regression might not work well. (2) Another method is to regularize the features of subjects from different sites respectively. Although this method may work well on other datasets, it is not suitable for ADHD-200 samples because datasets from some sites have only normal controls or few ADHD patients. We can use this method only when we abandon these datasets. (3) The third method is to train classifier on each datasets from different sites. However, this method, in fact, does not take advantage of the large samples of multi-site datasets. Moreover, the ADHD-200 test set has some samples from a new site, which have to be predicted using the model trained by the samples from other sites. In short, for complicated multi-site datasets such as the ADHD-200 Sample, the optimal solution is still pending and worth further investigation.

### Impacts of imbalanced class on classification

The ADHD-200 datasets have more TDC than ADHD patients (485 TDC vs. 281 ADHD). Such imbalanced datasets might cause bias of a classifier, making it more prone to classify samples to TDC. In fact, from the competition results released by the ADHD-200 Consortium, we found that for most participating groups, the number of subjects that were predicted as TDC was much more than the actual number of TDC in test set, which was one of the main causes of high specificity and low sensitivity in average (71.77% vs. 31.44%). A prediction system with an excessively imbalanced sensitivity and specificity is defective, which might at least suggest that the classifier is biased while training. We consider that we should pay more attention to other evaluation methods such as F-score or ROC graph rather than using only accuracy of classification.

In this paper, we use the selected datasets containing 404 TDC and 222 ADHD patients, in which the ratio of the number of TDC to that of ADHD patients is 1.82. Without special handling of this imbalance, we obtain the specificity of 73.13% and the sensitivity of 41.89% (using FC as features). However, in the competition, we use much less TDC than in this paper—only 517 subjects with 303 TDC and 214 ADHD patients. The ratio of the number of TDC to that of ADHD patients is 1.42. The competition results show that we obtained the specificity of 66.4% and sensitivity of 52.3%—the highest sensitivity and highest J-statistic (specificity + sensitivity – 1) in the contest. The reason we reduce the TDC used in the competition is that we want to prevent the impacts of imbalanced datasets as far as possible. We do not know the priori of the test set. Thus, we assume it as 50%, that is, the probability of a sample being TDC or ADHD patient is equal in the test set. However, you can also assume the priori as the same of training set (as most participants did). In fact, the actual number of TDC is slightly more than that of ADHD patients (the ratio is only 1.21, much less than that in training set). Thus, we consider that imbalanced class of training set and almost balanced class of test set might be one of the main reasons that cause the mean specificity much more than the mean sensitivity. The results of this paper also demonstrate this trend, that is, when using more imbalanced datasets, we obtain a more imbalanced specificity and sensitivity, and the impacts of imbalanced datasets on classification are different for different kinds of features. However, we do not do any further experiments about this discovery so that it is yet a hypothesis.

There are a number of solutions to the problem of imbalanced datasets at both the data and algorithm levels. At data level, different forms of re-sampling is commonly used, such as random oversampling with replacement, random undersampling, directed oversampling, directed undersampling, oversampling with informed generation of new samples, and combinations of the above techniques. While at the algorithm level, common solutions include adjusting the costs of classes, adjusting the decision threshold, one-class learning, etc. A special issue with respect to this problem provides an overview of these methods (Chawla et al., 2004). However, because we did not know the class distribution of the test set prior to the competition, we could not apply any of these methods to solve the problem in the competition.

### Conclusion and limitation

We have presented our classification framework for ADHD prediction. Four kinds of feature are extracted from the brain images and used for training classifiers. A hybrid feature selection method is applied before training SVMs in order to prevent the overfitting problem and reduce the computational complexity. We have used a nested CV method to tune the hyper-parameters of classifiers and evaluate the performances of our method, which can yield unbiased estimation of classification method. In addition to using a single feature, we also employ MKL to integrate multi-modal features. Our experiments show that MKL using multimodal features can yield better classification results for ADHD prediction.

Worth mentioning, this study has several limitations. The first is that we do not take into account the phenotypic information such as gender or age. The information may also contribute to the classification, as shown in the competition results of the University of Alberta. Another limitation of our framework is its performance on the imbalanced datasets. We believe that the imbalanced class of datasets affects the classification results, and when taking it into consideration, a classifier can achieve a better balance of its performances on sensitivity and specificity.

### Conflict of interest statement

The authors declare that the research was conducted in the absence of any commercial or financial relationships that could be construed as a potential conflict of interest.
